# How Hazards Turn Into Disasters: Perspectives of Emergency Responders

**DOI:** 10.1111/risa.70288

**Published:** 2026-06-11

**Authors:** Arkaprabha Bhattacharyya, Nazli Yonca Aydin, Tina Comes

**Affiliations:** ^1^ Resilience Lab, Faculty of Technology, Policy, and Management TU Delft Delft the Netherlands; ^2^ Systems Engineering, Faculty of Technology, Policy, and Management TU Delft Delft the Netherlands; ^3^ Decision Theory & ICT, Faculty of Technology, Policy, and Management TU Delft Delft the Netherlands

**Keywords:** cascading impact assessment, disaster and emergency management, disaster risk, fuzzy cognitive mapping, interdependence modeling

## Abstract

Natural hazards like floods, storms, or earthquakes turn into disasters if they hit vulnerable communities and societies. In policy and academia, this understanding has led to a surge of models and risk reduction policies that aim to reduce vulnerability and strengthen resilience. However, it remains unclear which vulnerabilities are the most important, and what stakeholders in different contexts prioritize. To address this gap, this article identifies critical exposure, vulnerability, and coping capacity factors, elicits their priority among emergency responders from different contexts, and analyses their perceived interdependences to understand their cascading potentials. To do that, we conducted a stakeholder survey with experienced disaster and emergency management professionals around the world. The results are used to analyze the perceived relationships between the priority factors via a fuzzy cognitive map. The professionals identified the level of preparedness, exposure to hazard, risk and crisis communication, community engagement, and disaster risk financing as the most important factors. The results show that the most catastrophic disasters are perceived to be caused by a combination of multiple factors and their interdependences. It was also found that practitioners thought that active civil protection agencies and available disaster risk financing have the greatest potential to prevent disasters.

## 1 Introduction

Fuelled by climate change and growing vulnerabilities, the impacts of disasters across the globe continue to rise. As communities and policy‐makers grapple to prepare for increasingly severe and frequent storms, floods, or wildfires, vulnerability and resilience have become central to disaster risk reduction and climate adaptation (Birkmann et al. 2022; Cutter et al. 2013). Today, there is no shortage of frameworks to measure resilience and vulnerability at different scales like community (Fran H. Norris et al. 2008), urban (Meerow et al. 2016), or societal (Birkmann et al. 2022), and in different sectors such as infrastructure (Francis and Bekera 2014), social (Saja et al. 2019), environmental (Folke 2006), or economic (Birkmann et al. 2022). Accordingly, there is a range of disaster risk reduction policies and methods that aim to address different facets of vulnerability and resilience.

At the same time, there is a discussion on how and how far disaster risk reduction policies and metrics for vulnerability and resilience need to be contextualized (Sanne et al. 2021; Weichselgartner and Kasperson 2010; Weichselgartner and Pigeon 2015). Acknowledging that local coping structures, norms, cultures, and risk profiles play a pivotal role for resilience and disaster risk reduction, we set out to investigate which of the many potential factors determining resilience and vulnerability are perceived as crucial across different contexts by experienced stakeholders.

Given the localization and complexity of the disaster domain, we asked professional disaster responders, emergency managers, and critical infrastructure providers to (1) prioritize different factors that drive disasters’ impacts across the categories of exposure, vulnerability, and coping capacity; and (2) to map out the perceived interdependences between these components. As we focus on vulnerability and resilience, this article takes a hazard agnostic approach (Trump et al. 2025) to capture the understanding of local emergency managers on key processes and priorities that can be tested empirically across possible hazards.

On the basis of this motivation, this article aims to answer the following research questions:

Which factors do stakeholders identify as key to disaster impact across the categories of exposure, vulnerability, and coping capacity? How do the identified factors influence each other leading to their cascading potentials?

To address these questions, we started by reviewing the existing literature to identify a list of factors that have been shown to influence disaster outcomes. Next, we conducted a stakeholder survey between May 2024 and June 2024, where we asked the survey participants to prioritize the most important factors and to indicate how they influence each other. We collected responses from 177 participants who have managed different types of disasters in different countries and contexts. The collected responses were aggregated through a fuzzy cognitive mapping (FCM) approach to derive a network of perceived interdependences. This network represents the collective understanding of the stakeholders on these factors and the interdependences among them. The network was utilized to understand the cascading potentials of vulnerability and resilience determinants.

The research results contribute to the body of knowledge as follows: First, we demonstrate the collective perceptions of disaster managements professionals on different exposure, vulnerability, and coping capacity factors and their relative importance in determining the outcomes of disasters. Second, we analyze how their experiences shape their preferences and perceptions, which reflects how contexts determine the most crucial determinants of disaster outcomes. Lastly, mapping the perceived interdependences helps us understand the combinations of factors that lead to the most catastrophic disasters. Collectively, these three contributions help us to understand why certain hazards turn into high‐impact disasters and in which contexts.

## 2 Conceptual Background

### 2.1 Hazards and Disasters

The terms “disaster” and “hazard” are often used interchangeably to refer to events like floods, storms, earthquakes (Alexander 2018; Bronfman et al. 2019; Cui et al. 2021; Raschky and Weck‐Hannemann 2007; Tan et al. 2024; Ward et al. 2020). The United Nations Office for Disaster Risk Reduction (UNDRR) recognizes hazards as the underlying processes or phenomenon that can be caused by natural events or human actions, and disasters as the disruptions caused when hazards interact with a vulnerable system that has insufficient coping capacity (UNDRR 2017a). This distinction implies that although hazards may be unavoidable, disasters are not. They emerge from the complex interaction between natural events and human systems.

### 2.2 Dimensions of Disaster Risk

The literature distinguishes three dimensions that explain why hazards turn into disaster: hazard and exposure, vulnerability, and coping capacity (De Groeve et al. 2015). Each dimension has different characteristics and has been conceptualized as discussed in the following:

***Exposure*** represents the situation of people, infrastructure, and other assets in a hazard prone area (UNDRR 2017b). Exposure is typically assessed by identifying the “elements at risk” (such as people or assets (United Nations 2015)) in hazard prone areas. To this end, research conventionally overlaps hazard zone predictions from hazard simulation models with administrative boundaries or population data (Zuzak et al. 2022).

***Vulnerability*** is defined as the susceptibility of a system to disruption by an external shock (Ezell 2007). In contrast to exposure calculation, vulnerability is conceptualized separately for people and social systems, and physical systems, infrastructures, and assets. Physical vulnerabilities within built environment are often derived from simulation techniques (Bellè et al. 2022; Nofal et al. 2024) or via data analytics (Bhattacharyya et al. 2023; Casali et al. 2024; Lee et al. 2022; Yabe et al. 2021). Social vulnerability is typically calculated as a composite index that combines various socio‐economic indicators such as household income, poverty level, race and ethnicity, and education (Cutter et al. 2003; Fraser 2021; Mah et al. 2023).

**Coping capacity** has been defined as the “determinants of adaptive capacity” (Yohe and Tol 2002) by means of the available resources, skills, and opportunities to manage and mitigate any adverse consequences of a hazard event. As such, coping capacity is an umbrella concept that combines social/community, economic, infrastructural, and (emergency) governance aspects (Parsons et al. 2016).

### 2.3 Global Frameworks for Disaster Risk

To compare the risks for different regions and countries, global disaster risk frameworks have been developed and refined over time. In the first world conference of natural disaster reduction in 1994, the UN member states adopted Yokohama Strategy and Plan for Action for a Safer World (United Nations 1994) primarily on focusing on improving coping capacity to ensure faster recovery from disasters (Tozier de La Poterie and Baudoin 2015). The subsequent Hyogo Framework for Action 2005–2015 identified various gaps in the previous framework, particularly risk identification and reducing underlying risk factors (United Nations 2005). Despite these ambitions, the Hyogo framework did not identify the underlying risk factors (United Nations 2015). The subsequent Sendai Framework (2015–2030) conceptualized disaster risk as a combination of vulnerability, capacity, exposure of people and assets, hazard characteristics, and environment (United Nations 2015). Although the Sendai framework made the different components of disaster risk more concrete, it did not provide the measurable underlying factors that could help prioritize disaster risk reduction or preparedness interventions.

To address this gap in operationalizing disaster risk, the INFORM framework and Risk Index were developed (De Groeve et al. 2015). INFORM has increasingly gained prominence in both policy‐making and practice of disaster risk reduction (Marzi et al. 2021). INFORM expresses disaster risk as a function of three dimensions: hazard and exposure, vulnerability, and lack of coping capacity. The INFORM Risk Index's hierarchical structure breaks down these three dimensions into categories, which are further broken down into components that can be measured by specific indicators.

Although the INFORM index has been successfully evaluated in the context of climate change (Marzi et al. 2021), it does not consider the risks of unsafe buildings that are especially pronounced in the context of earthquakes, floods, and storms (Aydin et al. 2025; Bhattacharyya and Hastak 2024; Yazdani et al. 2010). In addition, as a global index, the INFORM framework operates at country scale. As such, it does not consider the capacities at the community level that have repeatedly appeared in the existing literature (Choi et al. 2019; Cutter et al. 2014; F. H Norris et al. 2008).

In sum, despite the proliferation of disaster risk reduction frameworks in policy and research, three gaps remain. First, there is a lack of consensus on which specific disaster risk determinants (DRDs) are most important in influencing disaster outcomes within or across different contexts. DRDs represent the critical and measurable elements that mediate the transformation of a hazard into a disaster. Second, it remains unclear how these DRDs influence each other. Although research has advanced in analyzing cascading impacts of disasters (Pescaroli and Alexander 2015), interdependencies among broader socio‐economic, environmental, and institutional determinants have received less attention. This gap is particularly problematic in preparing for high‐impact low‐probability (HILP) events, where complex interactions between determinants can lead to unforeseen cascading failures. Third, although context is recognized as pivotal for effective disaster risk reduction (Sirenko et al. 2024), most research presents of case studies in specific regions or contexts, or of global studies—like in the case of INFORM—that operate with fixed weights. What is missing is an overarching understanding of how different contexts shape practitioner perceptions of which determinants are crucial to mitigate and manage disasters effectively. This understanding is essential for developing strategies that can address both everyday disasters and HILP events across diverse settings.

These gaps highlight the need to investigate how disaster managers across different contexts perceive the importance of various DRDs, and how they understand the interdependencies between these determinants.

## 3 Research Methods

**FIGURE 1:**
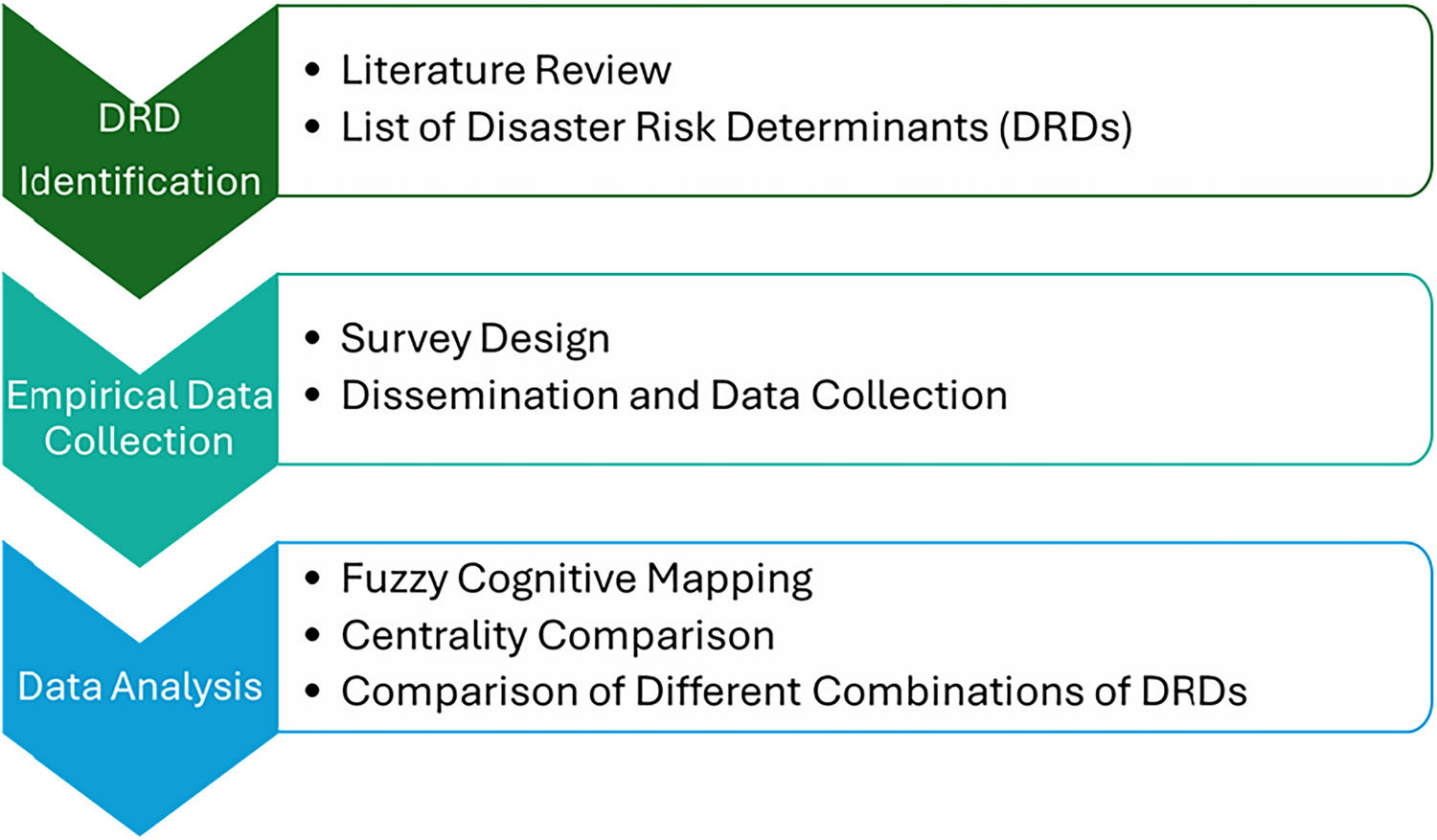
Methodological framework.

Our research was designed to address the identified research gaps through a systematic approach of (1) identification of DRDs, (2) empirical data collection, and (3) network analysis of perceived interdependencies. The methodological framework is shown in Figure 1. We adopted an exploratory approach that leverages the collective knowledge of experienced disaster management professionals to understand which determinants they perceive as most important and how these determinants interact. The following sections will explain each step.

### 3.1 DRD Identification

**FIGURE 2:**
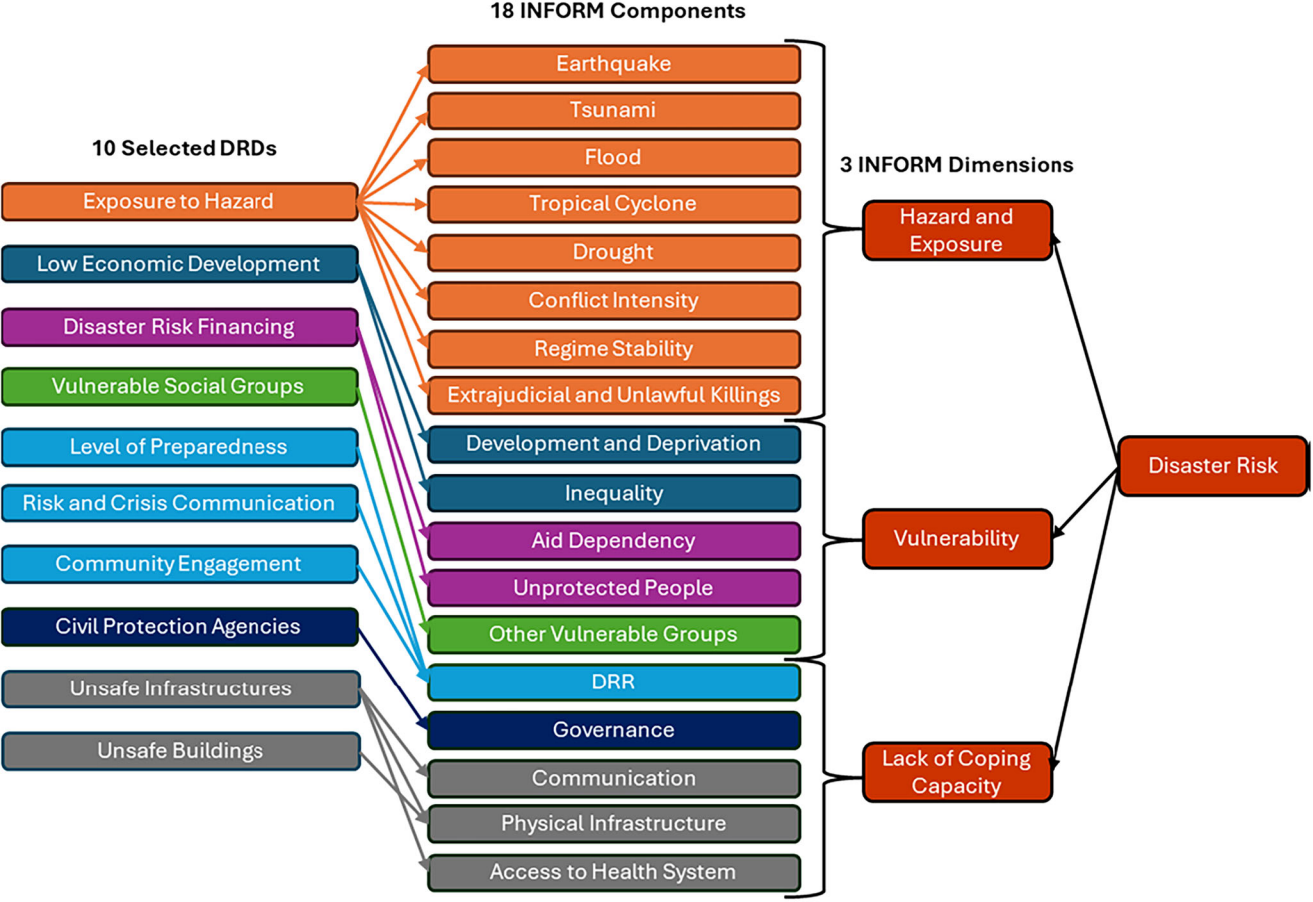
Synergies between the DRDs and INFORM framework.

We conceptualize disaster risk as a function of three dimensions: hazard and exposure, vulnerability, and coping capacity following the INFORM framework (De Groeve et al. 2015). To operationalize these dimensions into measurable DRDs, we lean on the INFORM framework for its prominence and popularity in DRR as shown in Figure 2 (right side presents the INFORM framework).

As our research methodology is based on an online survey of experienced emergency professionals, we sought to balance the inclusion of a large number of DRDs with the practical need to avoid survey fatigue and respondent overload. At the same time, we sought to maintain a balanced representation across all three dimensions; see left side of Figure 2.

We summarized 18 components of disaster risk in INFORM into 10 DRDs. The synergies between these 10 DRDs we chose and the 18 components in INFORM framework are also shown in Figure 2. These 10 DRDs are highlighted in different colors, and the corresponding components from INFORM framework follow the same color in Figure 2. This synthesis resulted in a set of 10 DRDs distributed across the 3 dimensions of disaster risk that formed the basis for our exploratory survey.

To account for potential gaps, we invited survey respondents to suggest additional DRDs. The majority of the respondents affirmed our list, strengthening the validity of our selection. This exploratory approach presents an initial step to test the broader influence of the selected dimensions, while recognizing that additional factors could provide further insight. The rationale for our choices is explained in the following subsections.

#### 3.1.1 Hazard and Exposure

The first dimension of disaster risk is hazard and exposure. The INFORM framework considers eight hazards separately. As this research is not focused on any particular hazard, that is, hazard agnostic (Trump et al. 2025), we considered “**exposure to hazard**” as one DRD. Exposure is a function of hazards’ magnitude and severity. Therefore, the magnitude or severity of hazard has not been considered another DRD.

#### 3.1.2 Vulnerability

We reorganized the five components of vulnerability within INFORM framework into three DRDs. The first two components of vulnerability in the INFORM framework are development and deprivation, and inequality. These two have been merged into one DRD called “**low economic development**” as inequality is often considered a component of low economic development (Aiyar and Ebeke 2020; Hartmann et al. 2017; Islam and McGillivray 2020). The next two components, aid dependency and unprotected people, in the INFORM framework reflect the ex post and ex ante components of disaster risk financing (World Bank Group 2016). Hence, these two have been merged into one DRD named “**disaster risk financing**.” The final component of vulnerability in INFORM framework, other vulnerable groups, has been kept separately as one DRD “**vulnerable social groups**,” which include children, elderly people, populations with disabilities, low‐income families, racial and ethnic minority groups, and population with low educational attainment (Cutter et al. 2012).

#### 3.1.3 Lack of Coping Capacity

Lack of coping capacity in the INFORM framework is a combination of five components, which have been redistributed into six for this research. First, we divided the component disaster risk reduction in INFORM framework into three DRDs “**level of preparedness**,” “**community engagement**,” and “**risk and crisis communication**” to distinguish capacity‐building from awareness‐raising actions. Preparedness is defined as the knowledge and capacities developed by governments, response and recovery organizations, communities, and individuals to effectively anticipate, respond to, and recover from the impacts of likely, imminent, or current disasters (UNDRR 2017c). Risk and crisis communication aim to create a shared understanding of risk among all involved stakeholders through coordination, collecting and disseminating information, and planning for a crisis and crisis management (Heath and O'Hair 2020). Community engagement refers to the process by which organizations, institutions, or groups involve individuals and communities in decision‐making, planning, and implementation of projects or initiatives that affect their lives (World Health Organization 2020). Due to its significance in community resilience (Aldrich 2012), we have considered community engagement as a separate factor that influences disasters’ outcomes. The governance component in the INFORM framework has been reshaped into strong “**civil protection agencies**,” which are governmental agencies or organizations such as police, fire protection, rescue service, and military, responsible for coordinating and implementing measures to protect citizens, property, and the environment during emergencies, disasters, and crises. The remaining three components of lack of coping capacity in the INFORM framework have been divided into two DRDs named “**unsafe infrastructure**” and “**unsafe buildings**” to distinguish the scale of disruption at network‐level (infrastructure) and asset level (building).

### 3.2 Empirical Data Collection: Stakeholder Survey

To gather the data on priorities and perceived dependences, an online stakeholder survey was conducted. The target respondents were professional disaster responders, emergency managers, and critical infrastructure providers who have managed different types of disasters in different countries and contexts.

#### 3.2.1 Survey Design

The objective of the survey was to get the practitioners’ perspectives on (1) which DRDs are the most important and in which contexts, (2) how they influence disaster outcomes, which were defined as the number of people affected and the associated economic losses (UNDRR 2020), and (3) how the DRDs influence each other. The survey was distributed online through different networks. It was open between May 2, 2024 and June 18, 2024. Before circulating the online survey, necessary ethics approval from TU Delft was obtained (application number 4172). A preview of the survey questionnaire is available at https://rb.gy/qlzlv2.

To answer the first question, that is, which DRDs are the most important, we asked the survey respondents to select and rank the five most important DRDs that they thought had the highest influence over disaster outcomes parameters. Respondents were asked to choose the five most important factors and place them in the corresponding buckets without replacement; that is, one DRD cannot be placed in multiple buckets.

The next question was designed to understand how each of the 10 DRDs influenced disaster outcomes on the basis of the respondents’ knowledge and experience. We collected responses in fuzzy terms in two steps. First, we asked about the direction of influence: Whether a factor “increases,” “decreases,” or “does not influence” disaster outcome parameters. Then, we asked about the degree of influence: whether the influence is “weak,” “moderate,” or “strong.”

For the third question, that is, how the DRDs influenced each other, the responses were collected in the form of an adjacency matrix. The five important DRDs selected by the survey respondents were carried forward and placed along the rows and columns of an adjacency matrix **A**. An entry **A** *_ij_* represents how the DRD along row *i* influences the DRD along column *j*. Like the influence on disaster outcomes, a DRD's influence on another DRD, that is, **A** *_ij_* could only have one of those seven fuzzy values—“strongly decreases,” “moderately decreases,” “weakly decreases,” “does not influence,” “weakly increases,” “moderately increases,” and “strongly increases.”

#### 3.2.2 Dissemination and Data Collection

The survey was primarily distributed via emails and popular social media platforms such as LinkedIn and X (formerly known as Twitter). The distribution started on May 2, 2024 after obtaining the ethics approval from TU Delft. The “EU‐H2020 AGILE” project participants were asked to complete the survey themselves and share it within their networks and connections. Additionally, a flyer was developed and distributed by project participants who attended the Humanitarian Networks and Partnerships Weeks at Geneva between April 29, 2024 and May 10, 2024 and European Civil Protection Forum at Brussels between June 4, 2024 and June 5, 2024.

**FIGURE 3:**
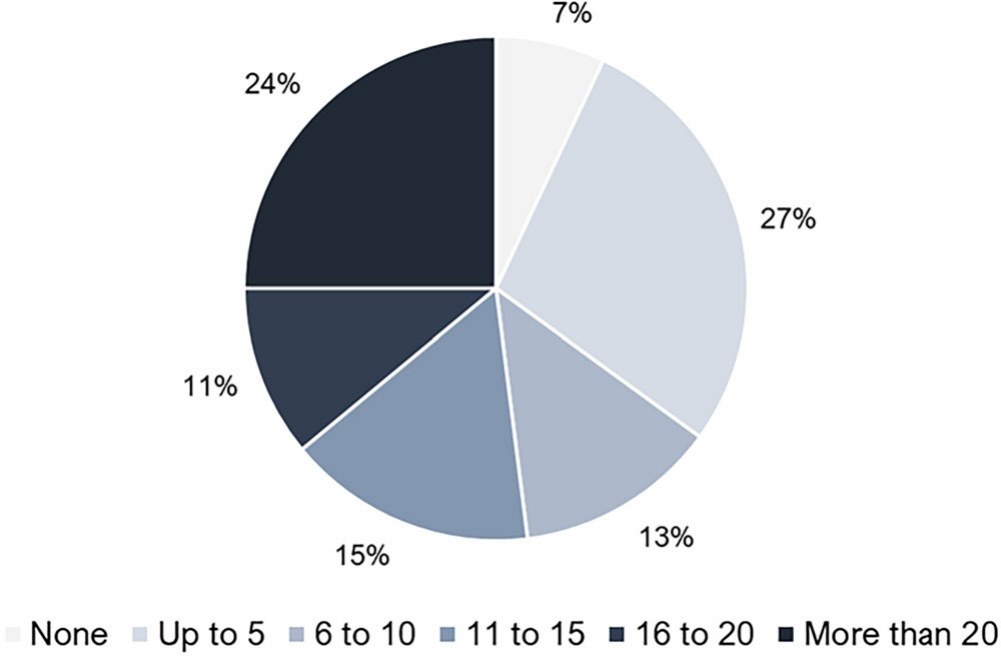
Years of experience of the survey respondents in disaster, crisis, and emergency management.

As a result, we collected 177 responses. The data collected through the survey is anonymized. After cleaning incomplete surveys and surveys that did not specify the five most important DRDs, we retained 104 responses for the analysis. Figure 3 shows the professional experience of the survey respondents in managing disasters. Around 63% of the respondents had more than 5 years of experience in managing disasters; thus, they can be considered experienced professionals. Almost a quarter of the respondents had more than 20 years of experience.

**FIGURE 4:**
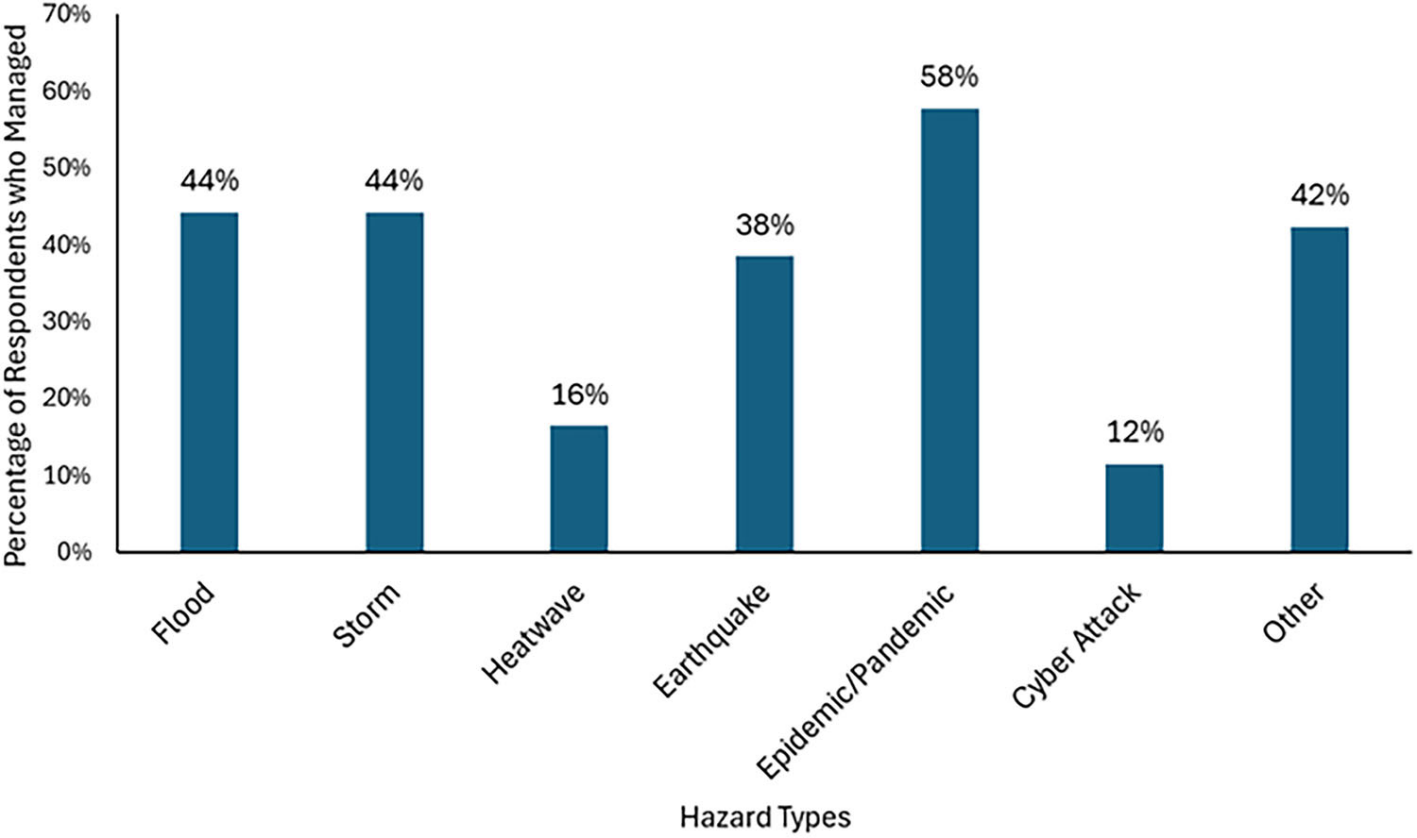
Types of disasters, crises, and emergencies managed by the survey respondents.

The survey respondents also managed different types of disasters, crises, and emergencies as can be seen in Figure 4. Nearly 58% of respondents have managed epidemics and pandemics, which may be a direct effect of the corona‐virus pandemic. In terms of natural hazards, the survey respondents have managed floods, storms, heatwaves, and earthquakes. A relatively small portion of the survey respondents had experience in cyber‐attacks. It is important to note that in this question, the respondents could select more than one option, and hence, the percentages do not add up to 100%. Nearly 42% of the respondents had managed “other” disaster types, including industrial accidents, wildfires, mudslides, tsunamis, volcanic eruptions, or airplane crashes.

**FIGURE 5:**
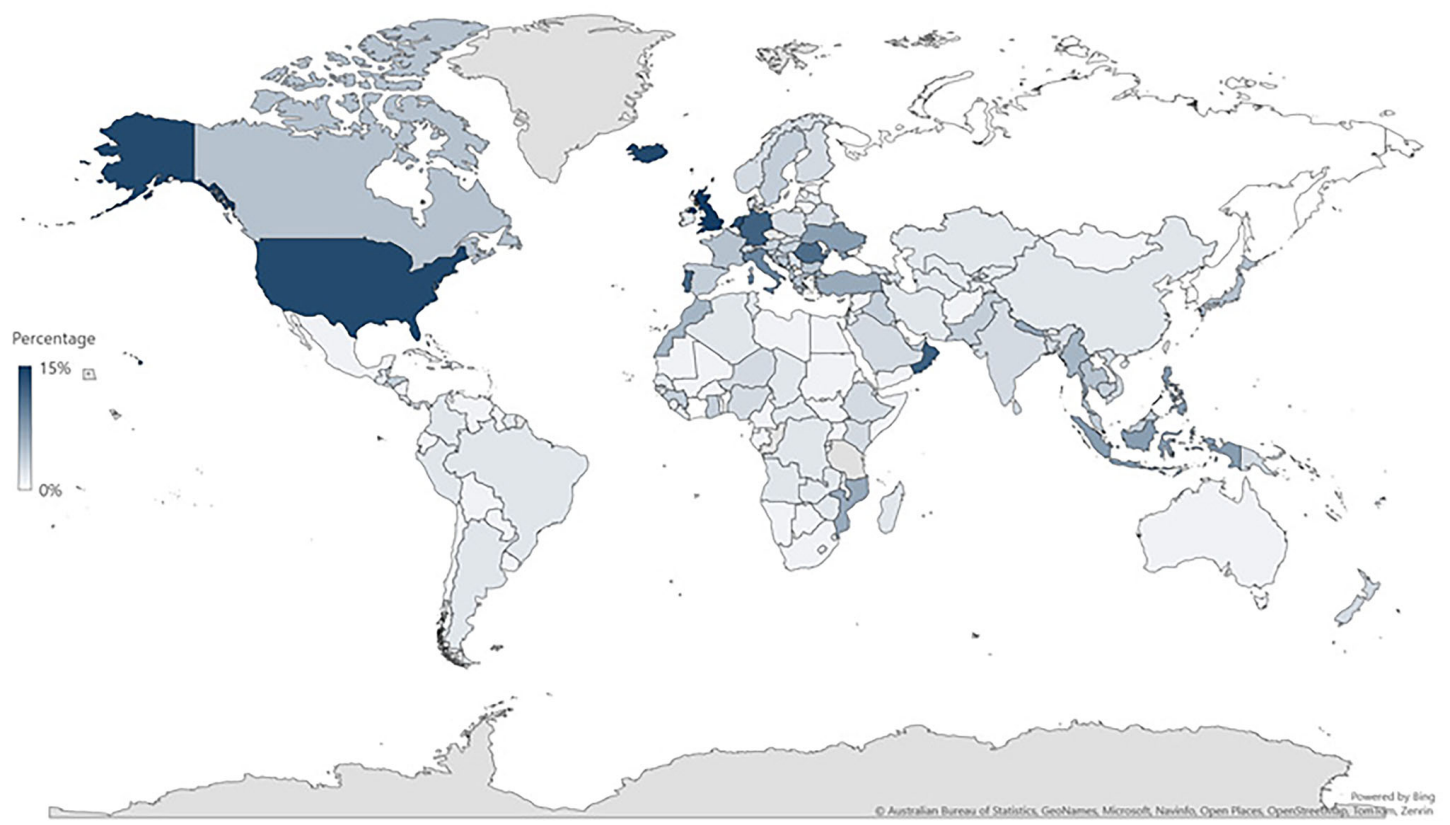
Countries where the survey respondents have managed disasters, crises, and emergencies.

In terms of geographical distribution, the survey respondents have managed disasters in 180 different countries out of the 193 UN‐recognized countries as shown in Figure 5. The top five countries are the United Kingdom (15% of the respondents), Iceland (14% of the respondents), the United States of America (14% of the respondents), the Netherlands (13% of the respondents), and Germany (13% of the respondents). As many respondents managed events in multiple countries, the number of countries (180) is higher than the number of respondents (104).

### 3.3 Data Analysis

As shown in Figure 1, three different types of analysis were performed with the collected data.

#### 3.3.1 Fuzzy Cognitive Mapping

To aggregate the fuzzy survey responses and develop a network of perceived interdependences, we used FCM, which was originally introduced by Kosko (1986) as fuzzy graph structure for explaining causal relationships. It has been used to model complex systems for decades. As it uses the knowledge and experience of the stakeholders living and working in the system, the cognitive map can be considered a collective representation of the system from the perspective of the stakeholders. As such, FCM is very popular in engineering (Bakhtavar and Yousefi 2018), environmental sciences (Poomagal et al. 2021), behavioral sciences (Poczeta et al. 2020), power system (Kuang et al. 2020), and so forth for its transparency and its ability to disentangle phenomena together with stakeholders. In this research, the purpose of the FCM is explanatory, meaning that we aim to understand how different combinations of DRDs lead to increased cascading effects.

FCM follows a graph structure, where the nodes are called the concepts and edges are called the connections. Helfgott et al. (2015) have categorized FCMs into two types: causal and dynamical. In the causal approach, the strength of a connection between concept A and concept B (directed from A to B) reflects the certainty about whether concept A causes concept B. In dynamical approach, the same connection would represent the magnitude of influence of concept A on concept B. The dynamical approach is suited for analyzing the propagation of effects of one concept on another (Helfgott et al. 2015), which is the purpose of the perceived interdependence modeling in this research. Therefore, a dynamic approach has been adopted in this research. In this approach, a concept can take any value, but it is often set between 0 and 1, where 0 indicates a concept is yet to be activated and 1 indicates it is fully activated. A connection typically has a value between −1 and 1, demonstrating the strength of influence of one concept on another.

**FIGURE 6:**
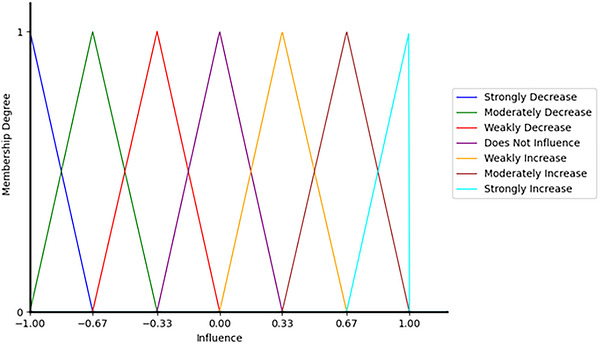
Membership function for the linguistic variable influence.

For this research, the concepts are the 10 DRDs and the two disaster outcome parameters, that is, the number of people affected and the associated economic losses from them. The survey gathered data on how these 10 DRDs influence each other and the disaster outcomes parameters in fuzzy terms. The corresponding membership functions are shown in Figure 6. For seven linguistic variables, we chose triangular membership functions following (Chen and Chiu 2021).

The fuzzy inputs were aggregated using the SUM method (Stylios and Groumpos 2004). The defuzzification of the aggregated response was conducted using the Center of Gravity (COG) method (Stylios and Groumpos 2004). Using the COG method, the expected value of a connection is calculated as Equation (1), where $w_{i}$ is the weight of each fuzzy linguistic variable, that is, the frequency of each linguistic variable based on the survey responses, $\mathrm{Are}a_{i}$ is the area of each triangular function as shown in Figure 6, and $x_{i}$ is the center of each triangular function:

(1) $$\mathrm{Value}\;\mathrm{of}\;a\;\mathrm{connection}=\frac{\sum_{i=1}^{7}w_{i}\times \mathrm{Are}a_{i}\times x_{i}}{\sum_{i=1}^{7}w_{i}\times \mathrm{Are}a_{i}}$$

Each DRD can influence the two outcome variables (perceived direct influence) and the remaining nine DRDs (perceived interdependences), resulting in a total 110 possible connections in the final graph. The FCM process was used to calculate the values of these 110 connections through aggregating survey responses using Equation (1). The perceived direct influence on disaster outcomes and the perceived interdependences have been presented separately in this article. The perceived direct influence has been presented in terms of a 10 × 2 matrix (10 rows for 10 DRDs and 2 columns for 2 outcome parameters). The perceived interdependences resulted in a network. The network is expressed as a 10 × 10 adjacency matrix, which is a tabular representation of the perceived network of interdependences among the DRDs. It basically shows the values of each connection (corresponding to the perceived interdependences) in the FCM in the form of a matrix, where DRDs are placed along the rows and the columns and an entry $A_{ij}$ in the adjacency matrix **A** represents how the DRD on row *i* influences the DRD on column *j*. As explained earlier, $A_{ij}$ can range between −1 and 1, where a negative entry reflects balancing effect and a positive entry reflects reinforcing effect of one DRD over another. An entry $A_{ij}$ represents how much the DRD on column *j* will change when the DRD in row *i* has a value of 1 due to their perceived interdependence.

#### 3.3.2 Centrality Comparison

The network of perceived interdependences was subsequently used to compare different centrality measures. Centralities reflect how connected or disconnected the DRDs are, which can gauge their cascading potentials. Moreover, on the basis of centralities, the relative importance of different DRDs in the network of influence can be compared. Three types of centralities have been computed. They are degree centrality, betweenness centrality, and eigenvector centrality (Schuerkamp and Giabbanelli 2024). Degree centrality measures the importance of a DRD within its immediate neighbors. It is computed as the sum of inward and outward centralities as shown in Equation (2), where, $\mathrm{ce}n_{d}\left(f_{i}\right)$ is the degree centrality of a DRD *i*, $w_{ji}$ is the weight of incoming connections from DRD *j* to DRD *i*, and $w_{ij}$ is the weight of the outgoing connections from DRD *i* to DRD *j*. As it can be noticed in Equation (2), degree centrality is the sum of in‐degree centrality ($\sum_{j=1}^{10}\left|w_{ji}\right|$) and out‐degree centrality ($\sum_{j=1}^{10}\left|w_{ij}\right|$). In‐degree represents how much a node is influenced by other nodes that are directly connected to it. Similarly, out‐degree centrality represents how much a node influences other nodes that are directly connected to it:

(2) $$\mathrm{ce}n_{d}\;\left(f_{i}\right)=\sum_{j=1}^{10}\left|w_{ji}\right|+\sum_{j=1}^{10}\left|w_{ij}\right|$$

Betweenness centrality measures a DRD's importance in bridging between other DRDs (Tchupo and Macht 2022). It is calculated as the sum of the shortest paths from DRD *s* to DRD *t* that go through the DRD *i*, as shown in Equation (3), where $\mathrm{ce}n_{b}\left(f_{i}\right)$ is the betweenness centrality of a DRD *i*, $\sigma_{st}$ is the number of shortest paths from a source DRD *s* to a target DRD *t*, and $\sigma_{st}\left(f_{i}\right)$ is the number of those paths that involve DRD *i*, that is, $f_{i}$:

(3) $$\mathrm{ce}n_{b}\;\left(f_{i}\right)=\sum_{\begin{array}{c} s,t=1\\ s\neq t\neq f_{i} \end{array}}^{10}\frac{\sigma_{st}\left(f_{i}\right)}{\sigma_{st}}$$

Lastly, eigenvector centrality measures the influence of a DRD. It is based on the importance of the DRDs it is connected to (Schuerkamp and Giabbanelli 2024). The eigenvector centrality of a DRD i is proportional to the sum of centralities of its neighbors and is calculated as the *i*th entry of the vector **x** as shown in Equation (4), κ is the largest eigen value of the adjacency matrix **A**, and **x** is the corresponding eigenvector:

(4) Ax=κx

#### 3.3.3 Comparison of Different Combinations of DRDs

On the basis of the network of perceived influence, we analyzed that combinations of DRDs are the most catastrophic. The analysis is based on the hypothesis that catastrophic disasters are not caused by a single DRD but due to a combination of several DRDs (Huque 2017; Liu et al. 2021). To do this, it has been assumed that a DRD can have three discrete states: non‐existent, intermediate, and activated. These three states are denoted by a DRD having the value of 0, 0.5, and 1, respectively. For example, the DRD exposure to hazard having a value of 0.5 will mean that the exposure is intermediate. As we have 10 DRDs and each DRD can only have three possible values, there are 3^10^, that is, 59,049 unique combinations possible among the 10 DRDs.

The cascading impact analysis starts with an initial vector $x_{0}$, which is a 1 × 10 vector showing the values of each of these 10 DRDs (0, 0.5, and 1). On the basis of the initial values of the DRDs, the cascading impact is calculated as Equation (5), where *C* is the final 1 × 2 vector showing the impact on the number of people ($C_{P}$) and economic losses ($C_{E}$), **A** is the 10 × 10 adjacency matrix capturing the perceived interdependences among the DRDs, and *D* is the 10 × 2 vector showing the perceived direct impact of DRDs on disaster outcomes shown in Table 2. The process is repeated for all 59,049 unique initial $x_{0}$ vector, and the corresponding effect on the number of people affected and the associated economic losses is estimated using the following equation:

(5) $$C=\left[\begin{array}{cc} C_{P} & C_{E} \end{array}\right]=\left[x_{0}A+x_{0}\right]\;D$$

Rather than serving for predictions, the outcomes of this analysis are meant for exploratory purposes showing how disaster outcomes change for different combinations of the DRDs based on their perceived interdependence. Before comparing the disaster outcomes parameters, that is, $C_{P}$ and $C_{E}$ for different combinations, they were normalized through a min–max normalization procedure. This step ensured that the ranges of $C_{P}$ and $C_{E}$ are limited to zero and one for the ease of comparison.

Out of the 59,049 combinations, we only focused on the combinations that led to highest values of the disaster outcome parameters, that is, the extreme outlier events. These outliers were classified as the events, where either of the two outcome parameters, that is, $C_{P}$ and $C_{E}$ were more than $\left(Q_{3}+1.5\times \mathrm{IQR}\right)$, where $Q_{3}$ is the third quartile and IQR is the interquartile range for the two parameters. We focus here especially on high‐impact events through segregating these outliers. We wanted to understand which combinations of DRDs are the most lethal based on the collective knowledge of the survey respondents.

## 4 Results

### 4.1 Priority DRDs

**FIGURE 7:**
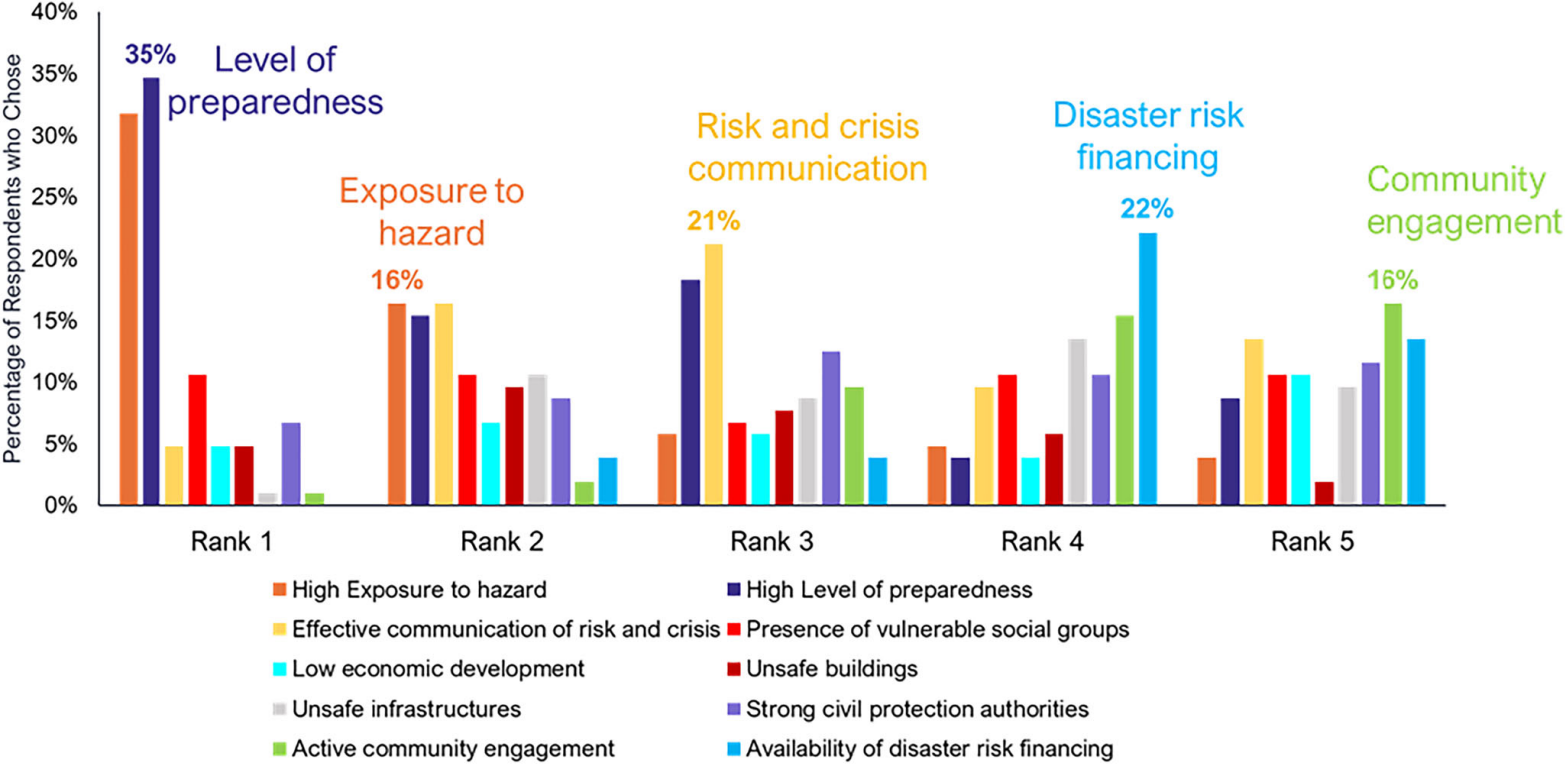
The most important DRDs determining disasters’ outcomes.

Figure 7 shows the five most important DRDs based on the survey. The figure shows the five buckets (rank 1–5, where rank 1 represented the most important DRD) and we asked the respondents to place one DRD in each bucket without replacement. The horizontal axis shows the distribution of different DRDs in each bucket, and the vertical axis shows percentage of respondents who chose a DRD in each bucket. For instance, nearly 35% of the respondents chose level of preparedness as the most important DRD and placed them in rank 1 bucket. Therefore, the level of preparedness has been identified as the most important DRD among the 10. For the second most important DRD, it was a tie between exposure to hazard and risk and crisis communication. As risk and crisis communication has appeared as the third most important DRD, to avoid repetition, exposure to hazard has been considered the second most important DRD. Disaster risk financing and community engagement have been selected to be the fourth and fifth most important DRDs, respectively.

In the survey, we operationalized level of preparedness through early warning systems, evacuation plans, temporary shelters, and so forth. Practitioners perceived these aspects as the most important contributors to disaster outcomes. Interestingly, disaster risk financing was not selected as the top priority, although it ranked fourth overall. In contrast, vulnerable social groups and strong civil protection authorities appear prominently in all five rankings. But they have not been picked as one of the five most important DRDs. Surprisingly, the level of economic development was also ranked low by the practitioners despite its importance in the literature (F. H. Norris et al. 2008).

Table 1 provides an overview on how the experience of managing different types of disasters in different countries influenced the choices of the survey respondents for the most important DRD. The table shows how many respondents (out of 104) selected a particular DRD as the most important one, and the average number of countries where they have worked (Figure 5), the average number of types of disasters they have managed (Figure 4) and the average years of experience they have (Figure 3). The table shows that for the top two choices, hazard exposure and preparedness level, there is a large difference in the average number of countries, where respondents worked. Respondents that prioritized high preparedness have worked on average in less than 4 countries, whereas for high hazard exposure, it was 8.5 countries. Moreover, low economic development has been prioritized by people who worked in many contexts (on average 9.6) versus unsafe buildings, infrastructures, or civil protection (all below 2 different countries). In other words, respondents that are familiar with a wider variety of contexts prioritize hazard exposure and economic development; those that are primarily experienced in their own country focus on the built environment and well‐functioning civil protection services, likely pointing to the differences in the Global North and South.

**TABLE 1:** How choices varied among the survey respondents (*N* = 104).

Most important DRD	No. of respondents who selected as top	Average no. of countries where they worked	Average types of disasters managed	Average years of experience
Level of preparedness	36	3.8	2.3	11.4
Exposure to hazard	33	8.5	2.6	11.6
Vulnerable social groups	11	3.5	2.8	11.5
Civil protection authorities	7	1.1	2.6	8.7
Risk and crisis communication	5	3.4	2.6	10.4
Economic development	5	9.6	3.8	13.0
Unsafe buildings	5	1.2	1.8	7.4
Unsafe infrastructures	1	2.0	2.0	18.0
Community engagement	1	1.0	5.0	13.0
Disaster risk financing	0	0.0	0.0	0.0

Abbreviation: DRD, disaster risk determinant.

The findings for types of disaster are less differentiated and point to a high specialization in terms of the types of disasters that people manage with most results being below three. The exceptions of low economic development (3.8 types of disasters) and community engagement (5 types) point to the fact that these aspects are important irrespective of the hazard.

Years of experience show a mixed picture. People with the least years of experience (7.4 years) selected unsafe buildings and active civil protection. If participants have more than 13 years of experience and worked on different disasters, then community engagement and economic development were prioritized; if the type of disasters they were managing was rather uniform, then the role of infrastructure was most prominent.

### 4.2 Direct Influence on Disaster Outcomes

As explained earlier, the perceived direct influence shows how a DRD directly influences the disaster outcome parameters. The aggregated responses on perceived direct influences are shown in Table 2. Positive influence or reinforcing effect indicates that prevalence of a *DRD increases* disaster losses. Table 2 shows that high exposure, high social vulnerability, low economic development, unsafe buildings, and unsafe infrastructure increase the extent to which disasters affect population and economy.

**TABLE 2:** Direct influence of disaster risk determinants (DRDs) on disaster outcomes.

DRDs	Influence on people affected	Influence on economic losses
High hazard exposure	0.69	0.61
High preparedness level	−0.57	−0.62
Better risk communication	−0.59	−0.55
Vulnerable social groups	0.55	0.38
Low economic development	0.36	0.22
Unsafe buildings	0.64	0.61
Unsafe infrastructure	0.65	0.66
Active civil protection	−0.61	−0.49
Active community engagement	−0.46	−0.44
Available disaster risk financing	−0.32	−0.37

Table 2 also shows the relative influence of different DRDs on disaster outcomes. For example, 1 unit increase in hazard exposure leads to 0.69 increase in the people affected and 0.61 increase in the economic losses. Among the five DRDs that increase disaster losses, high exposure to hazard has the highest influence on the people affected. Economic losses are primarily thought to be most influenced by unsafe infrastructures (Bhattacharyya et al. 2021).

The five remaining DRDs, that is, preparedness level, risk communication, civil protection agencies, community engagement, and disaster risk financing, reduce disaster losses. Therefore, these five DRDs have balancing effects on the disaster outcome parameters. Among them, active civil protection has the highest influence on reducing the number of people affected, whereas high preparedness level decreases the economic losses the most.

It can be noticed that the selection of the most important DRD (shown in Figure 7) and the direct influence on disaster outcomes (shown in Table 2) are not consistent. Although the level of preparedness is perceived as the most important DRD in Figure 7, its direct influence is not the highest on either of the two outcome parameters shown in Table 2. Contrarily, unsafe infrastructures, despite their low perceived importance in Figure 7, exert highest direct influence on the economic losses from disasters based on the collective understandings of the surveyed practitioners.

### 4.3 Unpacking Interdependencies

The adjacency matrix portraying the perceived interdependences among the DRDs is shown in Table 3. Out of the 100 elements in matrix **A**, the 10 diagonal elements are zeros as DRDs do not influence themselves. Out of the remaining 90, two others are zeros—$A_{57}$ and $A_{48}$. Out of the remaining 88 non‐zero entries in the adjacency matrix, 46 are positive, whereas 42 are negative. Among the positive interdependences, the influence of active civil protection agencies on the level of preparedness is the highest. Among the negative interdependences, the influence of the availability of disaster risk financing on unsafe buildings and infrastructures is the most pronounced. This reflects the importance of these two DRDs (active civil protection and disaster risk financing) in the eyes of the survey respondents. Not all the interdependences explained through the adjacency matrix **A** are causal in nature. For instance, the adjacency matrix shows that high exposure to hazard leads to enhanced level of preparedness against the hazard, which is not necessarily causal but can be indirect. In the example, high exposure may lead to frequent disaster experience, and, in turn, to higher investments into preparedness.

**TABLE 3:** Adjacency matrix.

	Hazard exposure (1)	Preparedness level (2)	Risk communication (3)	Vulnerable social groups (4)	Economic development (5)	Unsafe buildings (6)	Unsafe infrastructure (7)	Civil protection (8)	Community engagement (9)	Disaster risk financing (10)
High hazard exposure (1)	0.00	0.30	0.16	0.53	−0.28	0.24	0.38	0.10	0.21	0.36
High preparedness level (2)	−0.42	0.00	0.39	−0.18	0.35	−0.34	−0.37	0.43	0.33	0.30
Better risk communication (3)	−0.34	0.52	0.00	−0.29	0.37	−0.48	−0.37	0.47	0.42	0.26
Vulnerable social groups (4)	0.48	−0.16	0.04	0.00	−0.25	0.42	0.30	0.00	−0.04	−0.08
Low economic development (5)	0.37	−0.02	−0.17	0.27	0.00	−0.23	0.00	−0.29	−0.17	−0.42
Unsafe buildings (6)	0.50	−0.25	−0.10	0.07	0.17	0.00	0.35	−0.17	−0.25	−0.02
Unsafe infrastructure (7)	0.49	−0.27	−0.09	0.55	−0.42	0.44	0.00	−0.22	−0.29	−0.17
Active civil protection (8)	−0.34	0.61	0.39	−0.17	0.22	−0.63	−0.39	0.00	0.55	0.31
Active community engagement (9)	−0.18	0.52	0.38	−0.24	0.42	−0.31	−0.14	0.42	0.00	0.10
Available disaster risk financing (10)	−0.32	0.29	0.33	−0.21	0.38	−0.83	−0.83	0.46	0.18	0.00

### 4.4 Finding the Spider in the Web—What Are the Crucial DRDs That Highly Impact Exposure, Vulnerability, and Coping Capacity?

With the adjacency matrix, the centralities of the DRDs were estimated to compare their relative importance to the network of perceived interdependences. The centrality measures of the 10 DRDs are shown in Table 4, where the centralities are expressed as ranks to facilitate comparison. The DRD with the highest centrality measure is ranked the lowest.

**TABLE 4:** Disaster risk determinants (DRDs) ranked based on centrality measures.

	Ranks based on different centrality measures			
DRDs	In‐degree centrality	Out‐degree centrality	Betweenness centrality	Eigen vector centrality
High hazard exposure	2	7	3	2
High preparedness level	4	4	8	4
Better risk communication	9	3	6	10
Vulnerable social groups	7	10	1	6
Low economic development	5	8	3	5
Unsafe buildings	1	9	5	1
Unsafe infrastructure	3	5	8	3
Active civil protection	6	2	8	8
Active community engagement	8	6	1	7
Available disaster risk financing	10	1	6	9

These centrality metrics can be viewed as indicators of policy leverage and systemic interconnectedness on the basis of the collective perceptions of experienced disaster management professionals. These metrics identify where policy interventions should be made for the most impactful outcomes, how system‐wide improvements can be monitored, and what factors impact policy implementation.

High out‐degree centrality identifies the DRDs that have the most influence over other DRDs (Özesmi and Özesmi 2004). In Table 4, disaster risk financing has the highest out‐degree centrality. Therefore, it acts as the primary policy lever, in line with previous studies that have identified proactive disaster risk financing as a critical driver for mitigating disaster risk (Coetzee et al. 2023; Linnerooth‐Bayer and Hochrainer‐Stigler 2015). Table 3 shows that disaster risk financing can impact multiple DRDs via pathways such as upgrading infrastructure, hiring civil protection staff, or launching risk communication campaigns.

Conversely, nodes with high in‐degree and eigenvector centrality represent systemic dependency. These nodes are highly susceptible to the changes in the other DRDs (Özesmi and Özesmi 2004). They reflect where the consequences of failed policies in other DRDs ultimately manifest. Table 4 shows that unsafe buildings have the highest in‐degree and eigenvector centralities. From the adjacency matrix in Table 3, we observe that the three DRDs that influence unsafe buildings the most are lack of disaster risk financing, inactive civil protection, unavailability of risk and crisis communication. This is in line with the literature showing that failed policies in these DRDs manifest in terms of unsafe buildings (Aydin et al. 2025; Bhattacharyya and Hastak 2024; De Janvry et al. 2016).

The betweenness centrality metric highlights the bridges within the network (Jiang et al. 2026). In a policy context, these DRDs act as critical pathways. The high betweenness centralities of vulnerable social groups and community engagement suggest that if these social dimensions are neglected, even robust technical or financial interventions remain isolated and fail to scale. This finding confirms the need to strengthen social capital to effectively disseminate risk reduction efforts throughout the population (Aldrich 2012; Aldrich and Meyer 2015).

The observed connections in the network operate through direct functional pathways that explain how one factor leads to another. On the basis of the network of collective perceptions of experienced disaster management professionals, disaster risk financing serves as the essential first step because it provides the resources needed to strengthen the built environment and support social programs. Community engagement acts as the vital bridge that brings these high‐level resources and technical plans down to the local level. This connection ensures that safety measures are used and accepted by the public in their daily lives.

### 4.5 Understanding Cascading Potential

**FIGURE 8:**
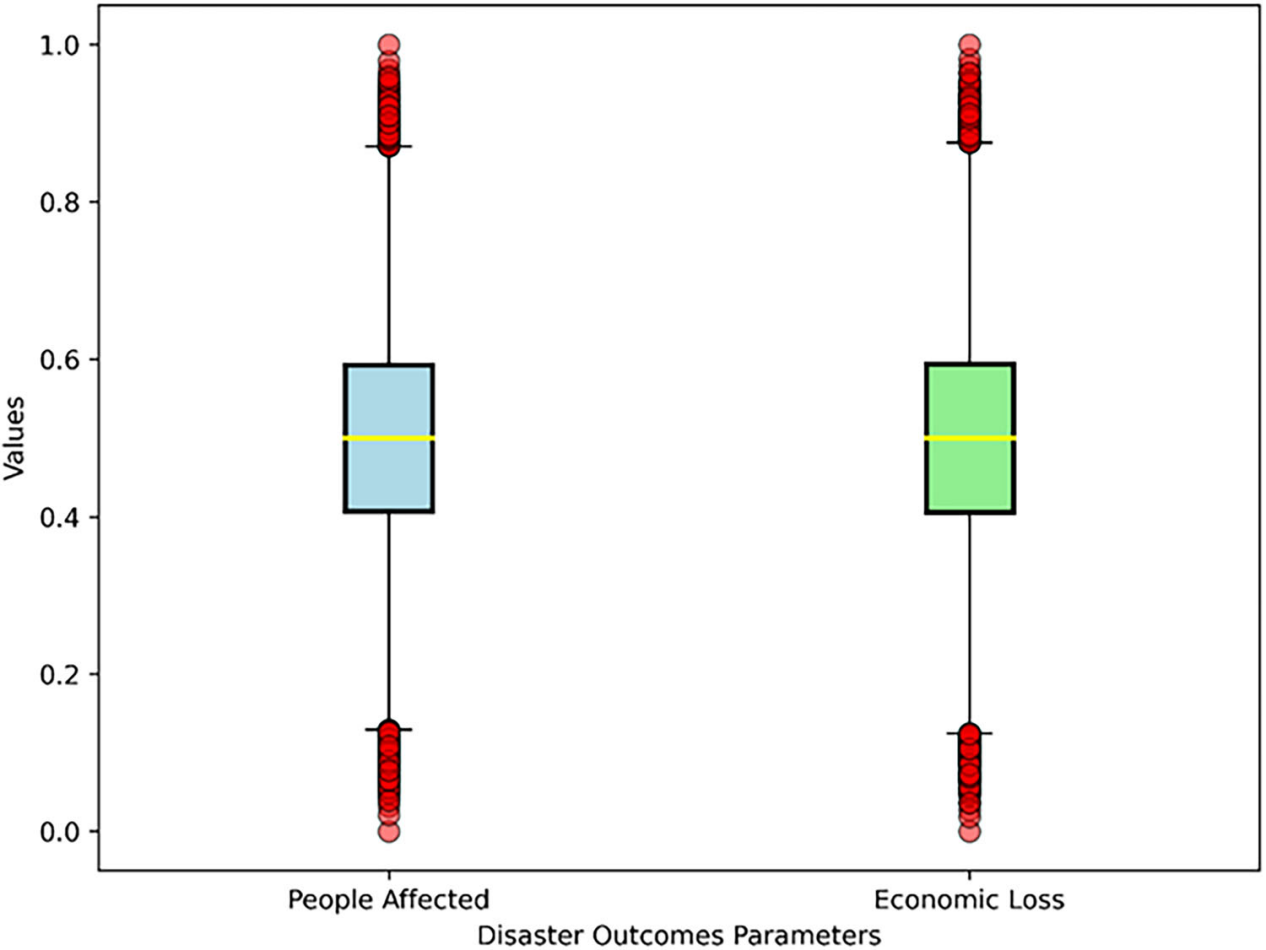
Boxplots of disaster outcomes parameters for different combinations of DRDs.

Next, we tested how different combinations of these DRDs influence the disaster outcomes. Figure 8 shows the boxplots for the two outcomes parameters for different combinations of the DRDs. The boxplots look very similar due to the very high correlation (0.99) between the two outcomes parameters.

As mentioned in Section 3.3.3, we conducted further analysis on the outlier events that signify disasters with catastrophic impacts. In Figure 8, the outliers are marked as red dots. The outliers on the upper whiskers of the boxplots are particularly of interest as they reflect the combinations of the DRDs that have extreme potentials of catastrophe. Out of the 59,049 combinations, 124 combinations resulted in one of the outcome parameters in the upper outlier zone. Further analysis has been limited to these outliers.

The combination that produced the highest number of people affected and economic losses has all five reinforcing DRDs shown in Table 2, that is, high hazard exposure, presence of vulnerable social groups, low economic development, unsafe buildings, and unsafe infrastructures activated (i.e., value of 1) and all five balancing DRDs, that is, high level of preparedness, risk and crisis communication, active civil protection agency, active community engagement, and availability of disaster risk financing non‐existent (i.e., value of 0). It is logical that the combination, where all reinforcing DRDs are prevalent, and all balancing DRDs are absent, will yield the highest values of the disaster outcome parameters due to the linear nature of Equation (5).

**FIGURE 9:**
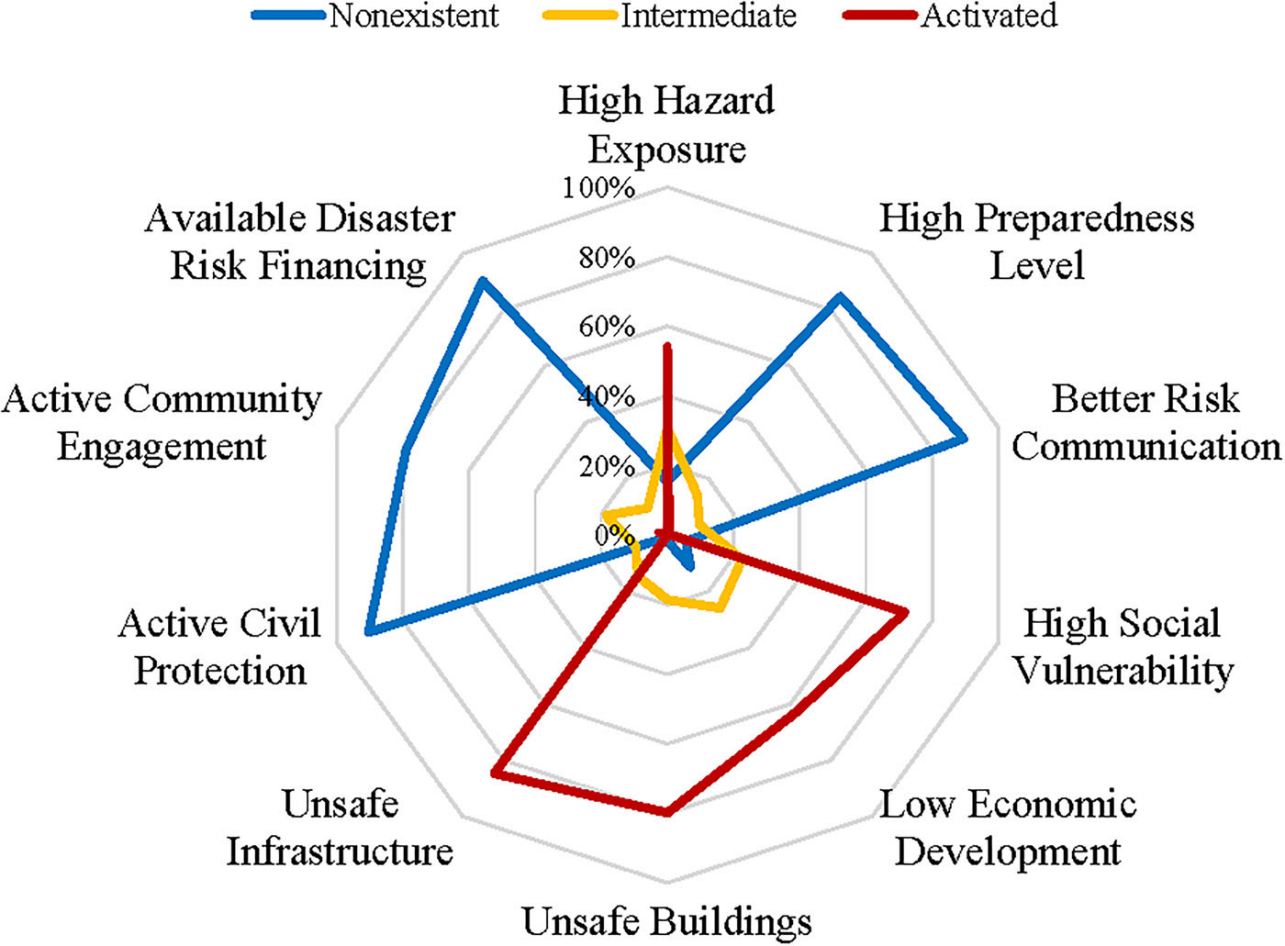
Proportion of DRDs among the outlier events.

Figure 9 demonstrates how the 10 DRDs are distributed across the 124 outliers. The figure is an extrapolation of the mental models of the practitioners and thus gives insightful information on how they perceive the root causes of catastrophic disasters. Importantly, non‐existent balancing DRDs are more prevalent than activated reinforcing DRDs. This indicates that the balancing DRDs that mitigate disaster risk are perceived as more important determinants of the magnitude of a disaster than reinforcing DRDs that amplify disaster risk. It is consistent with their preferences of mitigating DRDs as more important influencing disaster outcomes as evident in Figure 7. It also shows the importance of the roles of civil protection agencies and disaster risk financing in their eyes as none of the 124 instances in Figure 9 had activated civil protection agencies and available disaster risk financing. As the surveyed respondents can be considered a part of the civil protection community, it can be argued that they perceive their profession plays a fundamental role in determining the magnitude of a disaster. Moreover, the prevalence of activated unsafe infrastructures and unsafe buildings in Figure 9 indicates that the practitioners believed that majority of catastrophic disasters are caused by inadequate capacity in the built environment.

What is more insightful is that none of these 124 combinations is caused by any single DRD, but catastrophic outcomes are always caused due to the combination of more than one DRD, confirming work on cascading effects and systemic risk (Comes et al. 2022; Renn and Lucas 2021). This signifies that disaster risk reduction initiatives need to be designed to address multiple DRDs simultaneously as major disasters are caused by a combination of multiple DRDs. In the current practice, disaster risk reduction is often focused toward reducing exposure by designing and constructing disaster resilient infrastructures. Although their importance cannot be ignored, this research shows that experienced professionals believe ensuring adequate coping capacity, particularly active civil protection and available disaster risk financing, helping communities in disaster response and recovery, can mitigate the risks of catastrophic disaster losses more than traditional measures. Similarly, active civil protection agencies exert influence by providing the institutional framework and technical expertise necessary to bridge the gap between high‐level policy and local action. These agencies function as the operational backbone of the network, ensuring that theoretical risk communication and building standards are translated into tangible safety measures. Simultaneously, disaster risk financing shifts the system from a reactive to a proactive state by providing the liquidity required for infrastructure hardening and housing retrofitting. Thus, it reduces the inherent vulnerability of populations before a hazard strikes (Bhattacharyya et al. 2024). The high out‐degree centrality of these two DRDs confirms their role as primary drivers within the network. By providing both organizational coordination and the necessary capital, they create positive feedback loops that strengthen systemic resilience and prevent hazard events from escalating into catastrophic disasters.

## 5 Limitations

The primary methodological limitation of this study is the geographic concentration of the sample, with a majority of respondents having primarily worked in Global North countries. As the study aimed to capture a broad collective perception rather than a regional comparison, no weighting or stratification was applied. However, the resulting FCM and the ranked priorities may reflect risk management paradigms specific to high‐income contexts and potentially limit their generalizability to the Global South. For instance, the high importance assigned to disaster risk financing and active civil protection agencies reflects systemic reliance on formal financial markets and centralized state institutions that may not transfer to other contexts. As shown in Table 1, respondents who worked in more countries shifted their priorities toward economic development and exposure. This suggests that practitioners who are more likely to have worked in lower income settings hold a different mental model about which DRDs are most important.

In Global South contexts, formal mechanisms of *disaster risk financing* and *active civil protection* may be less accessible, as functioning formal insurances, capital markets, banking systems, sustained emergency services, or state institutions with legitimacy to translate policy into local action may be constrained. Traditionally, informal social safety nets, networks in the diaspora, or local community leadership (Ruslanjari et al. 2024; Surtiari et al. 2024) have sought to fill the resulting gap. Therefore, *community engagement* and addressing low *economic development* may emerge as structural levers in the Global South context rather than intermediaries.

These considerations caution against the direct translation of the rankings to low‐income settings or contexts with weak financial or state institutions. Future research can apply regional sampling strategies, ideally in a co‐design process with practitioners from the Global South to test whether, and in which ways, the identified priorities shift across governance contexts.

Second, we had to aggregate the 18 INFORM components into 10 key DRDs to ensure survey feasibility. This aggregation has resulted in broad DRDs like unsafe infrastructures and vulnerable social populations. It is, therefore, not possible to analyze the relative importance of different infrastructure sectors such as energy, health, and others. Similarly, vulnerable social populations also comprise different groups such as poor, disabled, seniors, children, racial and ethnic minorities, and others. The aggregation of these groups into one DRD did not allow us to analyze their relative importance.

As explained earlier, not all the relationships shown in the adjacency matrix are causal in nature. Ideally an adjacency matrix should only contain causal relationships. However, a number of relationships identified in this research are co‐existent or indirect. Overall, the adjacency matrix captures the collective understanding of the disaster management professionals and that can be expected to contain certain biases. Another example of that bias can be noticed in the most important DRD identification, which is evident from the difference of insights in Figure 7 and Table 2. In Figure 7, level of preparedness has been identified as the most important DRD. On the other hand, in Table 2, exposure to hazard and unsafe infrastructure has the highest influence (in absolute terms) on the number of people affected and the economic losses, respectively. Lastly, we only used three discrete levels for analyzing which combinations of DRDs are catastrophic. In reality, the DRDs are continuous in nature and thus can have any value.

## 6 Conclusion

Disasters due to natural hazards are increasing in frequency and severity across the globe. The distinction between hazards and disasters is an essential paradigm shift that encourages us to think of disaster risk as something that can be managed. On the basis of this premise, we conducted this research to identify why hazards turn into disasters. We have answered this question based on the collective understandings of experienced disaster management professionals. For that, we conducted a stakeholder survey, where we received responses from 177 experienced professionals, who have managed different types of disasters in 180 countries. The professionals identified the level of preparedness, exposure to hazard, risk and crisis communications, community engagement, and disaster risk financing as the most important DRDs that influence the extent of disaster damages. Further, we utilized their experiences and knowledge to develop a network that displays how these DRDs influence each other, and the disaster outcomes based on their perceptions. This network has led to several important insights on how disaster management professionals view the interdependence among the DRDs influencing disasters’ outcomes and their cascading potentials.

First, the survey respondents thought catastrophic disasters are always caused due to the prevalence of multiple DRDs rather than a single one. Hence, disaster risk reduction initiatives should adopt integrated approaches that address combinations of risk determinants simultaneously rather than targeting isolated factors.

Second, the practitioners see the DRDs that *mitigate* disaster risk (level of preparedness, risk and crisis communication, disaster risk financing, community engagement) as more important than the ones that *amplify* disaster risk (exposure to hazard) in determining the magnitude of disasters as evident from their choices of five most important DRDs shown in Figure 7. Therefore, practitioners think that DRR should emphasize mitigation measures focusing on capacity building rather than the typical approaches that reduce disaster risk and exposure.

Third, disaster management professionals think that the absence of active civil protection agencies and inadequate disaster risk financing mechanisms *always* lead to catastrophic disasters. Unsafe infrastructures and buildings, on the other hand, are the most important sources of physical vulnerabilities leading to catastrophic disasters. According to the respondents, DRR should prioritize robust civil protection and risk financing, while improving safety standards for infrastructures and built environment.

These insights provide a better picture into the mental models of practitioners in disaster management. As we have explained throughout the article, the outcomes should be used for exploratory purposes and are not suitable for making predictions as they are not based on empirical data. In future, the outcomes of the research could be verified and complemented by integrating insights from empirical evidence on these DRDs, which can then be used for predictive purposes and counterfactual testing. Additionally, the aggregated DRDs, such as vulnerable social population, could be disaggregated to understand relative importance the underlying components making up those DRDs.

## Conflicts of Interest

The authors declare no conflicts of interest.

## Data Availability

All data and Python scripts used to conduct this study are available on request from the corresponding author.
